# The TAS Test project: a prospective longitudinal validation of new online motor-cognitive tests to detect preclinical Alzheimer’s disease and estimate 5-year risks of cognitive decline and dementia

**DOI:** 10.1186/s12883-022-02772-5

**Published:** 2022-07-18

**Authors:** Jane Alty, Quan Bai, Renjie Li, Katherine Lawler, Rebecca J. St George, Edward Hill, Aidan Bindoff, Saurabh Garg, Xinyi Wang, Guan Huang, Kaining Zhang, Kaylee D. Rudd, Larissa Bartlett, Lynette R. Goldberg, Jessica M. Collins, Mark R. Hinder, Sharon L. Naismith, David C. Hogg, Anna E. King, James C. Vickers

**Affiliations:** 1 Wicking Dementia Research and Education Centre, University of Tasmania, Hobart, Australia; 2 School of Medicine, University of Tasmania, Hobart, Australia; 3 Royal Hobart Hospital, Hobart, Tasmania Australia; 4 School of Information and Communication Technologies, University of Tasmania, Hobart, Australia; 5 School of Psychological Sciences, University of Tasmania, Hobart, Australia; 6 Healthy Brain Ageing Program, University of Sydney, Sydney, Australia; 7 School of Computing, University of Leeds, Leeds, UK

**Keywords:** Dementia, Ageing, Artificial intelligence, Computer vision, Screening, Movement analysis, kinematics, finger tapping, dual-task

## Abstract

**Background:**

The worldwide prevalence of dementia is rapidly rising. Alzheimer’s disease (AD), accounts for 70% of cases and has a 10–20-year preclinical period, when brain pathology covertly progresses before cognitive symptoms appear. The 2020 Lancet Commission estimates that 40% of dementia cases could be prevented by modifying lifestyle/medical risk factors. To optimise dementia prevention effectiveness, there is urgent need to identify individuals with preclinical AD for targeted risk reduction. Current preclinical AD tests are too invasive, specialist or costly for population-level assessments. We have developed a new online test, TAS Test, that assesses a range of motor-cognitive functions and has capacity to be delivered at significant scale. TAS Test combines two innovations: using hand movement analysis to detect preclinical AD, and computer-human interface technologies to enable robust ‘self-testing’ data collection. The aims are to validate TAS Test to [1] identify preclinical AD, and [2] predict risk of cognitive decline and AD dementia.

**Methods:**

Aim 1 will be addressed through a cross-sectional study of 500 cognitively healthy older adults, who will complete TAS Test items comprising measures of motor control, processing speed, attention, visuospatial ability, memory and language. TAS Test measures will be compared to a blood-based AD biomarker, phosphorylated tau 181 (p-tau181). Aim 2 will be addressed through a 5-year prospective cohort study of 10,000 older adults. Participants will complete TAS Test annually and subtests of the Cambridge Neuropsychological Test Battery (CANTAB) biennially. 300 participants will undergo in-person clinical assessments. We will use machine learning of motor-cognitive performance on TAS Test to develop an algorithm that classifies preclinical AD risk (p-tau181-defined) and determine the precision to prospectively estimate 5-year risks of cognitive decline and AD.

**Discussion:**

This study will establish the precision of TAS Test to identify preclinical AD and estimate risk of cognitive decline and AD. If accurate, TAS Test will provide a low-cost, accessible enrichment strategy to pre-screen individuals for their likelihood of AD pathology prior to more expensive tests such as blood or imaging biomarkers. This would have wide applications in public health initiatives and clinical trials.

**Trial registration:**

ClinicalTrials.gov Identifier: NCT05194787, 18 January 2022. Retrospectively registered.

**Supplementary Information:**

The online version contains supplementary material available at 10.1186/s12883-022-02772-5.

## Background

The Lancet Commission [1] has described dementia as “*the greatest global challenge for health and social care in the 21st century.*” The progressive brain degeneration and associated loss of cognitive function have devastating effects on quality of life for people with dementia and their families, and enormous economic impacts on health and social care systems. The worldwide prevalence of dementia is rapidly rising, driven by ageing populations, and current figures of 50 million people living with dementia globally are predicted to triple to more than 152 million by 2050 [2].

The World Health Organization’s key strategy to reduce dementia prevalence is through prevention [3]. Research indicates that up to 40% of dementia cases worldwide could be prevented or delayed by addressing modifiable lifestyle and/or medical risk factors such as physical inactivity, smoking and hypertension [1, 2]. To maximise the effectiveness of dementia prevention strategies, we urgently need readily-accessible population-level screening tools – to facilitate targeted interventions for high-risk individuals or those early in the disease course.

The most common cause of dementia is Alzheimer’s disease (AD), that clinically manifests as progressive loss of memory, language and other cognitive functions. The underlying brain pathology is characterised by accumulation of abnormal amyloid beta (Aβ) and phosphorylated tau (p-tau) proteins followed by neurodegeneration. New data show that silent and progressive brain changes occur 10–20 years before cognitive symptoms emerge, with two stages of AD occurring before dementia: preclinical AD and mild cognitive impairment (MCI) [4, 5]. This critical period of preclinical AD, when there are no detectable clinical symptoms, is the optimal time to intervene with targeted dementia prevention interventions early in the disease [1].

Positron emission tomography (PET) brain scans and cerebrospinal fluid (CSF) tests [6] can measure preclinical AD pathology but they are too invasive, specialist or costly to be used at the population level; currently they are only available in a small number of hospitals and rarely in low- and middle-income countries. Standard pen and paper cognitive tests lack sensitivity in preclinical AD [7]. However, over the past 3 years, a range of new blood-based biomarker assays have been developed that can quantify AD pathology with minimal invasiveness, including in the preclinical phase.

One of the most promising blood-based biomarkers is plasma p-tau181, as it has been shown that levels increase across the AD clinical continuum and correlate strongly with CSF and PET measures of AD pathology [8, 9]. Furthermore, antemortem plasma p-tau181 levels 5–8 years before death predict AD brain pathology at post-mortem, including in those with unimpaired cognition [10, 11]. Baseline plasma p-tau 181 levels in individuals with unimpaired cognition (as well as those with impaired cognition) are predictive of prospective cognitive decline and neurodegeneration [12]. In summary, the development of blood-based biomarkers is a major advance towards identifying preclinical AD in clinical and research settings, and plasma p-tau181 is considered both sensitive and specific to AD pathology. However, the practicalities and cost of obtaining a blood sample and accessing highly-specialist analytic equipment limit wide accessibility to most populations around the world, as well as for large-scale population intervention studies. Very few research centres have access to the specialist and expensive highly sensitive analytical infrastructure required to analyse the blood samples and each biomarker test typically costs at least $50–100 USD.

Thus, there remains urgent need for low-cost non-invasive and accessible tests that can pre-screen individuals for their likelihood of AD pathology prior to more specialist and expensive biomarker tests, or simply to identify individuals at higher risk for targeted risk reduction. A population-level screening test for preclinical AD would have wide applications in public health initiatives and clinical trials. Similar to screening programs for other chronic diseases such as diabetes, cancer and heart disease, a test that allows people to identify likely preclinical AD pathology would provide more time to reduce dementia risk before cognitive decline and before the brain degenerates; it would also enable opportunities for early recruitment to clinical trials. This would transform the effectiveness of dementia secondary prevention strategies in reducing dementia incidence.

There is a growing body of research that shows movement analysis is an effective method to identify the preclinical AD phase; several studies have shown that patterns of human movements change in preclinical AD and decline across the AD continuum. For more than a decade, research studies have found that slowed walking, especially when a dual motor-cognitive task was performed, predicted cognitive decline and dementia years later [13, 14]. However, the scalability of detailed gait analysis is limited by the need for wearable sensors, in person measures, or gait laboratories, and there are also substantial risks of falls.

More recently, several different types of studies have provided strong evidence that hand movement analysis is a sensitive biomarker of preclinical AD. First, functional magnetic resonance imaging (MRI) research has revealed that the human posterior cingulate cortex is specifically involved in control of hand movements [15] and this is highly relevant because the posterior cingulate cortex is known to be one of the first areas of the brain to show abnormalities in preclinical AD – in terms of both hypometabolism [16] and Aβ deposition [17]. This new imaging discovery accords with well-established single-unit microelectrode studies in monkeys that show high activity in the posterior cingulate cortex during forelimb self-paced tapping movements via projections to the primary motor cortex [18]. Taken together, these studies provide evidence that hand movement tests can detect the earliest pathological changes associated with preclinical AD.

Further, in a recent study of older adults with preclinical AD (defined by having elevated CSF Aβ indicative of AD, but with unimpaired cognition) Mollica et al. (2019) found that the speed and variability (a measure of rhythm) of simple finger tapping tests on a computer keyboard are abnormal in preclinical AD, and the *degree* of variability correlates with CSF Aβ levels [19]. These findings support earlier studies that determined hand reaction times correlate highly with CSF Aβ biomarker levels in preclinical AD [20] and progressively worsen over the AD continuum [20–22]. Additionally, it has been shown that even with unimpaired cognition ApoE4 carriers have been found to have delayed hand reactions compared to non-carriers, suggesting hand movements deteriorate at the very earliest stages of preclinical AD [20].

Based on this mounting evidence, and the wide accessibility of computers, including among older adults [23], we have devised TAS Test (or ‘Tasmanian Test’^1^), a new 20-minute online platform of digital motor-cognitive tests that are designed to be completed in people’s own homes without any specialist supervision. Specifically, TAS Test collects a range of hand movement data using standard computer cameras, keyboards and mouse-click data. It builds upon our work that found computer vision and other cutting edge artificial intelligence (AI) technologies can accurately measure hand movements using standard computer equipment [24–27], and that hand movements deteriorate with cognitive decline [28, 29].

For the first time, we will use AI video technologies in a health-related test. Similar technologies have already successfully automated cancer screening from static images (eg breast and lung from mammograms and radiographs, respectively), but we will analyse much richer video images, and collect these in the participant’s own home, or preferred remote setting. Our prior research shows that computer vision accurately measures hand movements [24, 25, 27, 30] and TAS Test combines these advanced technologies with hand-movement tests that are known to be affected in preclinical AD [19–22]. Using plasma p-tau181 test results as ground-truth, this novel remote method has high potential to reliably predict preclinical AD risk. In addition, it also includes computerized tests of cognition that are based on principles of other neuropsychological tests. This will allow exploration of motor-cognitive associations and the development of multi-modal predictive algorithms using a range of different types of data.

In this research, we therefore look beyond the current definition of dementia – a clinical syndrome of cognitive decline – to investigate the detection of AD from a new perspective: by focusing on hand movement analysis. This novel approach capitalises on recent discoveries about movement in preclinical AD and learns from historical neurology breakthroughs made by questioning definitions, particularly around the entwined relationship between movement and cognition*.* For example, Parkinson’s disease, defined as a pure movement disorder for almost 200 years, is now recognised as having ‘non-motor’ symptoms (such as loss of sense of smell, depression and cognitive decline) decades before movement decline. This paradigm shift has unlocked a whole new field of Parkinson’s disease research, producing new biomarkers, drugs and screening tools and greatly improving peoples’ quality of life. Similarly, we could transform AD prevention and research by analysing ‘non-cognitive’ brain functions impaired at the earliest stages of pathology.

There are several practical advantages to using hand movement as the core assessment of a population-level test - including the ease with which it translates across other countries, cultures and languages, and the speed and ease of assessment that can be performed while sitting in front of a home computer. With wide accessibility to computers, we envisage that TAS Test may be an inexpensive first-line population-level screening test that can identify high-risk individuals, including those in remote and rural communities, for further evaluation (including with blood biomarkers, where available) and/or risk modification.

### Aims and hypotheses

The aims of the study are to:

1. Develop and validate the optimal TAS Test protocol to detect preclinical AD and,
2. Validate TAS Test to prospectively estimate 5-year risks of cognitive decline and AD.

We hypothesise that, for adults aged 50 years or older, TAS Test will:

1. Detect preclinical AD (defined as positive blood p-tau181, normal cognition) with high sensitivity and specificity.
2. Estimate the 5-year risk of accelerated cognitive decline and dementia with high precision.

## Methods

### Aim 1

To develop and validate the optimal TAS Test protocol to detect preclinical AD.

#### Design

A cross-sectional observational research design will be used to develop TAS Test protocols and validate these against blood biomarker-defined preclinical AD.

#### Setting

Multi-centre recruitments will take place in Australia via established cohort studies at the University of Tasmania and the University of Sydney, aiming for 500 adults aged 50 years old or more. Participants will be assessed online at home (or their own preferred remote setting) or in the University clinical research facilities if they prefer. It is expected that most will complete the tests at home. They will use a laptop computer or desktop computer with a webcam and microphone. Participants who have not already provided a blood sample for their respective studies, will be invited to attend the University clinical research facilities to do so.

#### Participants

The participants will be recruited from the Tasmanian Healthy Brain Project (THBP) [31] at the University of Tasmania (UTAS) (ethics reference H0018265), and The Healthy Brain Ageing (HBA) Program [32] at the University of Sydney (protocol number 2019/271).

The THBP is a National Health and Medical Research Council (NHMRC)-funded, prospective cohort study that evaluates cognitive ageing in older adults who reside in Tasmania, Australia. Between 2010 and 2014, 556 participants aged 50 years and older were recruited (mean age 60 years) and underwent detailed neuropsychological and cognitive assessments, medical screening, and genetic tests at baseline, with detailed (4–6 hours) annual cognitive assessments for 4 years, then biennially.

The HBA is a specialist early intervention clinic and prospective cohort study that has recruited more than 1,000 participants aged 50 years and older (mean age 66 years) who have concerns about their cognition and reside in New South Wales, Australia. All receive detailed annual clinical phenotyping, including neuropsychological and medical assessments.

Participants from THBP and HBA will be invited to take part in the TAS Test study via email invitation or in person at a scheduled study visit. Inclusion criteria: Male and female adults ≥50 years old with unimpaired cognition. Exclusion criterion: Participants with impaired cognition, defined as declining cognitive trajectories measured by CANTAB Paired Associates Learning (PAL) total errors adjusted for age and sex, and a score > 1.5 SD above the mean total errors adjusted [33]. The rationale for excluding participants with impaired cognition is that, in this study, we aim to develop a test that discriminates participants with preclinical AD (unimpaired cognition and positive p-tau181) from those with healthy ageing (unimpaired cognition and negative p-tau181). Participants may withdraw from the study at any time and for any reason without any consequence to their ongoing participation in THBP or HBA. When an individual withdraws from the study, all the information collected to that point will be kept in the database for data analysis or withdrawal analysis.

#### Measurements

Each participant will complete TAS Test online at baseline with follow-ups 3 and 6 months later. They will also be invited to provide a blood sample at baseline for p-tau181 levels. The plasma p-tau181 levels will be measured using the ultrasensitive Single Molecule Array immunoanalyser (Simoa®, Quanterix) at the University of Tasmania. Previous research established p-tau181 > 1.81 pg/ml as sensitive and specific to preclinical AD, and highly predictive of AD risk (hazard ratio 10.9) in people with unimpaired cognition or MCI [9]. However, in recognition that the research field of blood-based biomarkers is rapidly evolving [34], and the threshold levels will depend on the assay used, we will use a cut-off value determined through an examination of the most current literature.

### TAS Test protocol and data extraction

#### Consent

The online consent form is presented to participants on the password protected secure TAS Test website. It lists relevant consent statements and participants must insert their name and date of birth at the bottom of the consent form before clicking the submit button to document their consent. Repeat consent is obtained each time TAS Test is attempted.

#### Instructions

The TAS Test online protocol presents a series of general instruction screens to ensure that the participant is correctly positioned in front of their computer in a well-lit and quiet room. The TAS Test software automatically detects whether the computer camera and microphone are functioning and asks the participant for permission to use these during the forthcoming tests. If the participant does not have a camera and/or microphone on their computer, or does not give permission for their use, they may still complete sections of the assessment protocol that do not require these.

There are five sections in the TAS Test protocol:

(i) Video hand movement tests
(ii) Keyboard hand movement tests
(iii) Visuomotor tests
(iv) Visuospatial ability
(v) Oro-motor and language abilities

### Video hand movement tests

A 5-second looped demonstration video of a researcher performing the required hand movements provides instructions before each new task. This is supplemented by written instructions that automatically appear on the screen and audio instructions that will be activated if the participant presses an ‘audio’ icon. For each task, the participant is instructed to hold their hands up so they can see them fitting inside green ‘data collection’ boxes that appear on the screen. A number of hand movement tasks, based on sensitivity to preclinical AD, or AD, in previous studies [19–22, 35] are tested in a fixed (non-randomised) order [1]: whole hand opening-closing in antiphase [2] finger tapping (FT) each hand separately at comfortable speed [3]; FT each hand separately at maximal speed [4]; FT both hands in-phase at maximal speed × 3 trials [5]; FT both hands anti-phase at maximal speed [6] FT both hands in phase at maximal speed with a cognitive task (counting backwards out loud from 100 i.e. dual task).

The dual motor-cognitive task has been included so we can calculate the motor cost, defined as any degradation in performance in the motor task (e.g. finger tapping) due to the additional demands of the cognitive task. People with low cognitive capacity or AD display greater dual-task motor costs than healthy age-matched controls and dual-task cost in older people with MCI is predictive of subsequent conversion to AD [36]. Audio recordings will be made during the video recording and specialised dictation software can interpret (cognitive) performance in terms of the number of correct responses and the number of errors.

Each trial is 10 seconds except for the 20 second dual motor-cognitive task. To prevent fatigue, participants initiate the start of each trial when they are ready and may take rests between trials. Participants may also stop part way through the protocol and then return to complete tests later as all interactions with the TAS Test protocol are time-stamped.

To extract the hand motor features from video data, we will develop a neural network-based computer vision method to detect and track hand keypoints (e.g. fingertip positions) in the frames of the captured video data and a model to calculate features from each individual finger tap in video data. We will use a sliding window approach to extract movement features selected by experts for analysing their correlation to preclinical AD (p-tau181 levels). We will explore an unsupervised approach using a deep network mapping directly from video clips (or keyboard-tapping data, see next section) to probability of preclinical AD. We anticipate this will give rise to discriminative spatiotemporal features in the hidden layers that could be visualised as an aid to understanding. Thus, human/supervised and agnostic/unsupervised deep learning methods will extract discriminative movement features as input data for the multivariable model (see Statistical Analysis section).

The following data will be extracted from the hand movement videos: mean tapping frequency, total tapping count, intra individual variation, mean inter tap interval, coefficient of variance of tapping frequency, coefficient of variance of amplitude, maximum speed, decrement on speed and decrement on amplitude. For the antiphase tasks, we will also measure bimanual coordination and phase shift between hands.

### Keyboard hand movement tests

Participants will be asked to complete a series of computer keyboard-tapping tasks “as fast and as accurately as you can” based on previous protocols [19, 37]. Specifically there are 8 keyboard tests in TAS Test comprising: spacebar tapping with the dominant hand for four blocks of 10 seconds tapping with 30 seconds rest periods between each block; dominant hand index finger tapping for 30 seconds alternately on two adjacent keys; dominant hand index finger tapping for 30 seconds over a defined sequence of three adjacent keys; right hand index finger tapping for 30 seconds across the keyboard between the ‘S’ key then the ‘;’ key; and left hand index finger tapping for 30 seconds across the keyboard between the ‘S’ key then then ‘;’ key. The last two tests are adapted, with permission, from the original The BRadykinesia Akinesia INcoordination (BRAIN) Tap Test developed by Noyce et al. [37] The BRAIN test was originally designed to measure motor performance in people with Parkinson’s disease. We adapted the test by instructing participants to perform the test only once with each hand (rather than two blocks of 30 seconds tapping) and using our own custom written software for data collection. All on screen instructions (Start signal, stop signal etc) and all keyboard tapping events are time-stamped in milliseconds. The following movement data will be extracted from the keyboard tapping data: tapping frequency, rhythm, intra-individual variation across repeat tasks, accuracy and delays in initiation and inhibition of movements.

### Visuomotor tests

#### Adapted version of Cats-and-Dogs test

The Cats-and-Dogs test used in TAS Test has been adapted, with permission, from the original test designed by Weil et al. [38]. The original Cats-and-Dogs test is an online test designed to detect visuoperceptual deficits in people with Parkinson’s disease; participants are shown a series of images, with each one showing either a cat or a dog, and some images skewed at an angle. There are 6 practice images followed by a test set of 16 images. Each image is presented on the screen for 450 ms, followed by a choice screen where the participant is asked to indicate whether the image shown was a cat or a dog.

In our adapted version of the Cats-and-Dogs test, we ask the participant to hold the computer mouse cursor over a ‘Start button’ at the bottom of the screen to trigger the display of each image. This modification allows additional measurements of the reaction time (time to release the start button), movement time (time to move from start button to cat/dog choice button) and latency (time to re-set the start button after the cat/dog choice button) in addition to whether the image is correctly identified (accuracy), image subject (cat vs dog) and image distortion (degree of skew distortion).

#### Reaction time tests

**Fig. 1:**
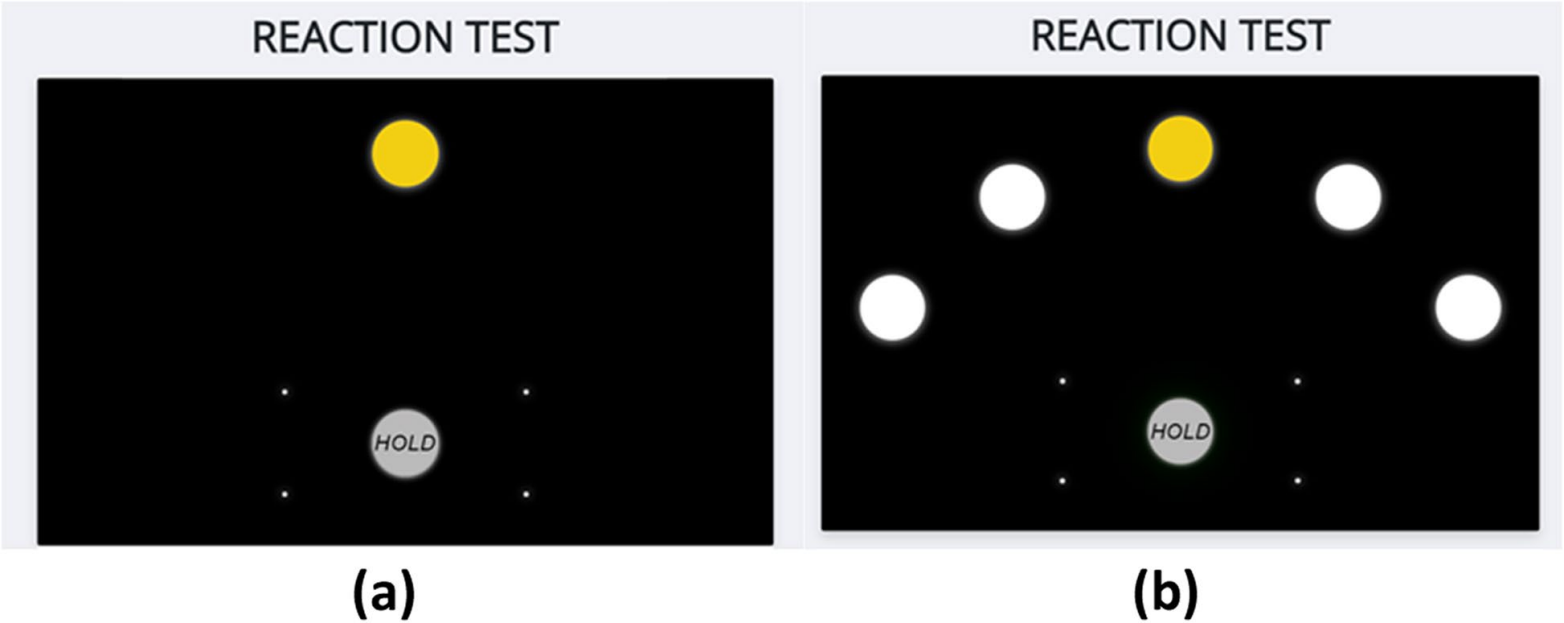
TAS Test Reaction Time Tests: **a**) simple choice test and **b**) five choice test

TAS Test includes two Reaction Time tests that measure the participant’s processing speed: a simple reaction time test (Fig. 1a) and a five-choice reaction time test (Fig. 1b). Each test has a practice phase of two trials followed by a test phase of five trials.

The participant begins each test by clicking and holding their cursor in the light grey circle at the bottom of the screen (Fig. 1). For the simple reaction time test, there is one white circle in the centre of the screen. The participant is instructed to click on the circle as soon as it turns yellow. The circle will turn yellow after a variable, and random latency of 2, 3 or 4 seconds. Once the participant has achieved 5 out of 6 clicks correct in a row, or completed a maximum of 18 attempts, the protocol moves onto the five-choice reaction time test. In this test, five white circles are presented in the centre of the screen in a chevron formation. The participant will begin the test by clicking and holding their cursor in the grey circle. When one of the five white circles (randomly allocated) turns yellow after a variable duration (range 2–4 seconds), the participant needs to click the yellow circle as fast as possible. For each test, the participant will have to click on the grey circle to begin the next trial.

The data extracted from each test will include: an accuracy score, which is the total number of trials in which the response is recorded as correct; an error score, which is the total number of trials in which the response is recorded as incorrect (this will be split into errors of timing and errors of location); reaction time (time to release the start button), movement time (time to move from start button to yellow circle) and latency (time to re-set the start button after each trial).

### Visual and spatial working memory

#### Benson complex figure test

**Fig. 2:**
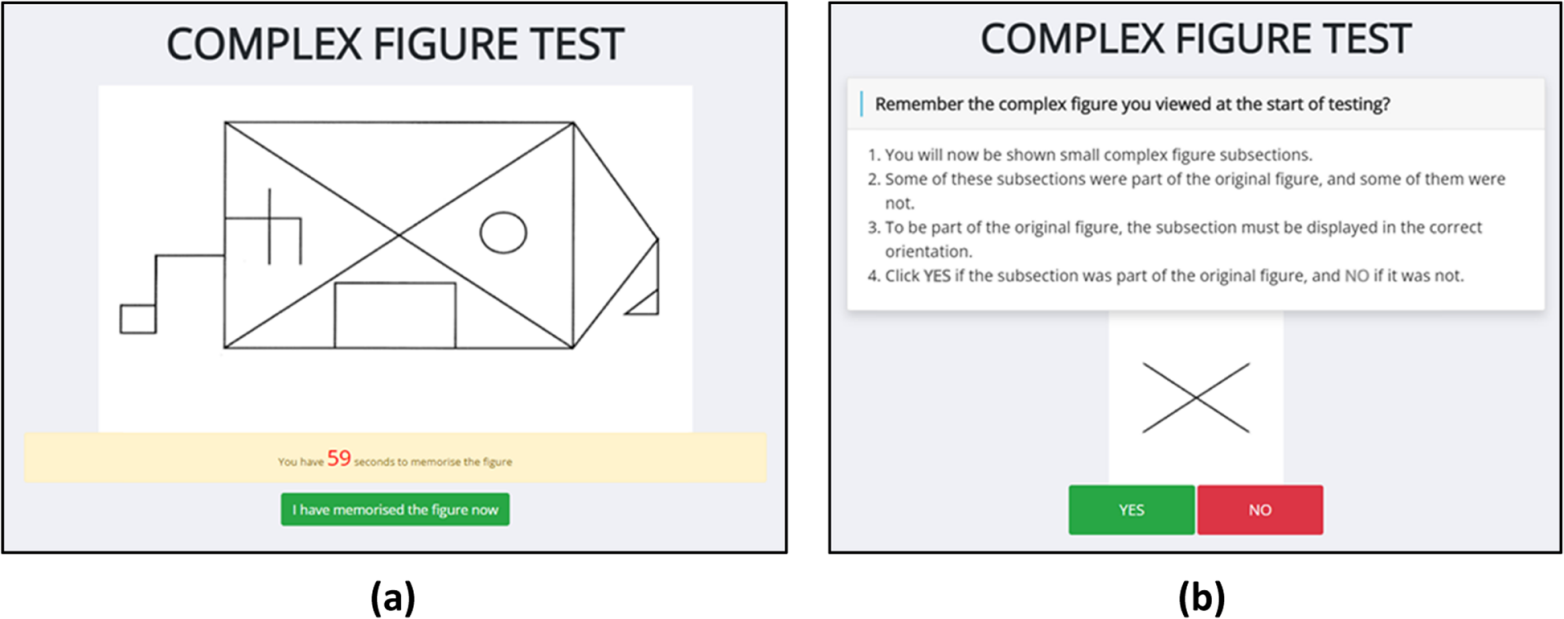
Benson Figure Test (**a**) Viewing phase to register the shape over a 1-minute duration and (**b**) delayed recall phase to identify whether a sub-section was part of the original figure

The Benson Complex Figure Test assesses a participant’s visuospatial ability; it is based on the Benson Figure, that was developed by Frank Benson as a simplified version of the Rey-Osterrieth complex figure [39] (Fig. 2) and has been adapted, with permission, for online use in TAS Test.

In the viewing phase, participants will be shown a complex image in its entirety and have up to 1 min to commit the complex figure to memory. Participants may click “I have memorised the figure now” before the one-minute period has ended, to move on to the next task. After completing the spatial span test (see next section) participants will be asked to recall if sub-sections, that are presented on the screen in a random order, were part of the original image or not. One at a time, 20 different subsections of the Benson Figure will be randomly shown and the participant will click “YES” if they think the sub section was part of the original image, and “NO” if they think it was not. Ten of the subsection images will be from the original image and ten of them will not. The modification for TAS Test is to present the participant with sub-sections of the original shape, rather than the original task whereby the participant draws the shape from recall.

The parameters extracted from the Benson Figure Test will include: duration of original viewing phase; time between viewing phase and recall phase; recall latency, which is the mean time it takes to respond; accuracy score which is the total number of image subsets that were correctly responded to; miss score, which is the total number of image subsets that were incorrectly responded to; recall test duration, which is the total time taken to complete the recall phase.

#### Spatial span

The Spatial Span test assesses a participant’s working memory in two phases, a practice phase and a test phase. It is based upon the Corsi Block-Tapping Task, in which a participant would mimic the order a researcher taps on a series of wooden blocks [40]. In this test, participants are asked to register a sequential series of coloured circles, that fill in outlines of circle shapes on the screen, and then immediately recall this sequence in the correct order.

**Fig. 3:**
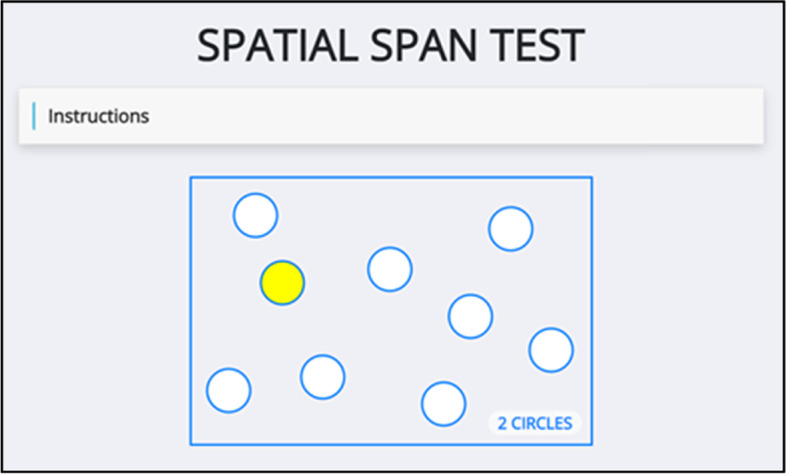
Spatial Span test; the participant is shown a sequence of yellow circles and then asked to repeat the sequence by clicking on the circles. The length of sequence increases each time a sequence is correctly recalled, up to a maximum of 9 circles

The participant will be shown the correct sequence first, then asked to repeat the sequence, by clicking on the individual circles; see Fig. 3. In both modes (practice and real), the number of circles in the sequence is increased from a level of 2 at the start of the test to a final level of 9. In the bottom right-hand corner of the screen there is a notification of how many circles will be coloured in the task, for example “2 CIRCLES”. Circles are only coloured yellow for 500 ms, with a gap of 750 ms between the previous coloured circle going blank and the next circle becoming coloured. There are three possible sequences at each level, but as soon as the participant passes a sequence at each level (e.g. the 3-circle sequence) they will immediately progress to the next level (e.g. 4-circle sequence), not necessarily performing all three sequences at each level. As soon as the participant clicks an incorrect circle, they are informed of their error and given a second (and third if necessary) opportunity to attempt the test again with the same number of circles but a different sequence. If all three sequences at any level are completed unsuccessfully, the test will terminate.

The parameters calculated include: spatial span length score, which is the longest sequence correctly recalled by the participant; total miss errors, which is the total number of times the participant missed selecting the correct circle in the test phase; total usage errors which is the total number of times the participant selected an incorrect circle in the test phase; reaction time, which is the time between the last circle of the sequence being presented and the first circle of the recalled sequence being selected; inter-circle movement time; which is the time taken to move from one circle to the next in recalling the sequence and total sequence movement time, which is the time taken to complete recalling each sequence.

### Oro-motor and language abilities

It is well recognised that language impairment is an early feature of AD – with deterioration in particular in naming and verbal fluency. Lesser known, are potential correlations between the motor aspects of speech production and voicing and how these may relate to the preclinical phase of AD [41–44]. To capture a broad range of speech and voice in a short period of time, we have included a standard picture describing task. Participants are shown the ‘Cookie Theft’ picture from the Boston Diagnostic Aphasia Examination [41] and asked to verbally describe it. This black and white line-drawn image conveys a complex scene of a woman and two children in a kitchen with interacting objects in which various activities are occurring. Participants are shown the following instructions: *“Below you see a picture of a scene. Please press the “Start recording“ button (below the image) and, in your own words, describe what you see in the most detail you can. Try to talk for at least a minute. When done, press “Stop recording“, and then “Submit””.* There is no time constraint on this task, although participants are encouraged to speak for at least 1 min. Participants' responses will be audio-recorded by the computer microphone for post-hoc analyses of their lexical, pragmatic, semantic and syntactic verbal proficiency using custom-written software programs. Deep learning approaches will be used to automate this process and to learn associated features.

### Further data collected

Data will also be collected on age, sex, years of education, English as first language, dominant hand, motor and cognitive symptoms, levels of pain and anxiety, and neurological diagnoses (Supplementary file 1). The following passive data will be collected without user input: log in time, log out time, user’s real-time mouse track, click actions on website internet browser (Chrome/Firefox/Safari etc). By collecting times of start/stop button clicking, we can understand how participants behave when they are using the application. For example, if a participant clicks start and stop several times, this means that the recording process may be difficult for participants to use. Time of day may influence performance. The following system-related data will be collected: Internet Protocol Version 4 (IPV4) address, browser information (name, version, product, manufacturer, layout), operating system, computer screen resolution, webcam information (resolution and frame rate). By collecting system information, such as browser information (version, product, manufacturer, layout), operating system (version, platform), camera resolution, video upload time, we can evaluate how different system environments may affect application administration.

### Aim 2

To validate TAS Test to prospectively estimate 5-year risks of cognitive decline and AD.

#### Design

We will undertake a prospective 5-year cohort study. We will invite 10,000 adults from a long-term cohort to complete online tests at home: (i) TAS Test every 12 months, and (ii) Cambridge Neuropsychological Test Automated Battery (CANTAB) [42] cognitive tests every 24 months. The prospective ‘high risk’ predictions of TAS Test will be validated against CANTAB, and clinically via in-person testing in a subsample of 300.

#### Participants

All eligible participants in The Island Study Linking Ageing and Neurodegenerative Disease (ISLAND) will be invited to take part in the TAS Test project. ISLAND is a 10-year public health initiative that launched in 2019 at the University of Tasmania (ethics reference H0018264). The project aims to recruit 20,000 people over 50 years of age who live in Tasmania, Australia by the end of 2022 and decrease their dementia risk; the detailed protocol is described in Bartlett et al. [43]. Nearly all assessments for the ISLAND project (except blood-based biomarkers that are collected in the research centre) are administered via an online portal where participants use a password to log in to their profile and complete a variety of assessments that are launched by the research team every few months. ISLAND participants complete extensive health questionnaires at baseline and will also be invited to complete TAS Test annually and CANTAB biennially, all online.

To date, over 13,500 participants have been recruited. CANTAB tests of cognitive function have been collected from approximately 3500 participants, TAS Test assessments from approximately 2200, and blood samples for the ApoE4 gene and blood biomarkers have been collected from approximately 2000. We can thus track cognition over time and identify people with scores indicative of cognitive decline as the sensitivity/specificity of CANTAB for classifying MCI and early dementia are 97/81% and 82/97% respectively [33].

The inclusion criteria for the TAS Test Aim 2 study are: ISLAND participants who have completed ≥2 TAS Test and ≥ 2 CANTAB tests. Exclusion criteria are: probable, or known, diagnosis of dementia or neurological disorders that impair hand function, speech or language function (e.g. stroke, Parkinson’s disease, collected through the baseline and interval ISLAND online health questionnaires). Participants will be classified as having ‘stable’ or ‘declining’ cognitive trajectories depending on PAL total errors at baseline (30 or more) and an increase of more than 10 total errors at follow-up assessments, as defined by Fowler et al. [44].

A sub-sample of 300 people will be selected from the main ‘TAS Test Aim 2’ cohort to attend the linked ISLAND Cognitive Clinic; this clinic provides facilities and expertise for gold standard clinical consensus diagnosis in a ‘one-stop’ interdisciplinary clinic where participants will have neuropsychological, and geriatrician or neurologist assessments, MRI brain scans, and ApoE4 tests. To select this cohort of 300 people for further face-to-face assessments from the larger cohort of up to 10,000 participants, we will invite 200 ISLAND participants (randomly selected) from those who have CANTAB scores (at the 48 month test point) indicative of MCI or AD – ie those with learning/working memory or attention cognitive deficits. A further 100 will be selected as age- and sex-matched controls from the Aim 2 participant cohort (using stratified random sampling) that comprises people with CANTAB scores indicative of unimpaired cognition.

#### Measurements

We will extract baseline demographics and health survey data, 12-monthly TAS Test data, and CANTAB tests at baseline, + 24, and + 48 months.

### Sample size

#### Aim 1

We estimate that 17% (or 85 individuals) in the cognitively unimpaired THBP & HBA cohorts will have p-tau181 in the preclinical AD range [45]. We have performed ROC curve analysis using open data from Mollica et al. [19]. This showed area under ROC curve (AUC) of 0.75 for a null linear model including age, sex, ApoE4, and years of education, but not finger tapping, to detect p-tau181 positivity in CSF. To develop the multivariable model, power calculation showed the sample size to compare a screening test with an area under the curve (AUC) > 0.90 against the null model would require 60 positive p-tau181 cases and 290 controls. Thus, we will use 350 in the development dataset (expected 60 [17%] preclinical AD cases) and 150 in the validation dataset (expected 25 [17%] preclinical AD cases) to test cut-offs from the development model. The PROBAST tool [46] confirmed these combined development and validation strategies lower the risk of bias.

#### Aim 2

Based on the age distribution of ISLAND participants, we project that 8.6% of participants currently aged 70–74 years (a cohort of ~ 1300 from the 10,000 recruited) will develop dementia, and more than 16% of the 700 participants currently aged 75+ years will develop dementia over the study duration [47, 48]. Thus at least 200 participants will meet diagnostic criteria for dementia or MCI. Our sample size calculation aims to ensure precise estimation of model parameters while minimising potential of overfitting. A sample of 300 including 100 dementia cases would ensure accurate prediction of the outcome proportion (33%) with a targeted confidence interval of 28–38%. To target a mean absolute prediction error (MAPE) < 0.05, as recommended by Riley et al. [49], a sample of 300 would be adequate for up to 6 predictors in a parsimonious, statistically determined risk prediction model.

### Statistical analysis

#### Aim 1

To develop the TAS test protocol and validate it against p-tau181, data from 350 participants with no cognitive impairment will develop the model. Data from the remaining 150 participants in this validation dataset will determine the model’s sensitivity and specificity in detecting preclinical AD. Replicates of plasma biomarkers will be assessed for outliers and then averaged. Hand-movement data will be summarised for each participant. Participants with unimpaired cognition and mean p-tau181 above a diagnostic cut off will be classified as ‘preclinical AD’. We will use multiple logistic regression to model the associations between movement features and p-tau181 positivity. Covariates may include age, sex, ApoE4 genotype, years of education, and handedness. As a secondary, more agnostic approach, we will use a deep learning approach to discover features in the video and keyboard movement data that map to p-tau181 positivity. To validate the TAS Test multivariable model, we will use cross-validation for model selection to avoid overfitting and bootstrap procedures to estimate model uncertainty. We will independently validate the model using the remaining 150 participants’ data. Receiver Operating Characteristic (ROC) curves will be plotted against the positive p-tau181 cut-off to assess the sensitivity and specificity of movement models to identify the preclinical AD stage. As TAS Test is intended as a screening test, the resulting curves will allow users to make informed choices about sensitivity and specificity that align with their research or health promotion goals.

#### Aim 2

To validate prospectively against cognitive decline, we will assess the sensitivity and specificity of TAS Test to predict cognitive trajectories (“stable” and “declining”) using ROC curve analysis. We will estimate 5-year cognitive decline/MCI/AD risk using baseline TAS Test data in a sub-sample of 300 people. Clinical diagnostic categories are: cognitively unimpaired, MCI, and AD. Covariates considered in the model will include age, sex, ApoE4, years of education, and handedness. We will use Bayesian multinomial logistic regression with regularizing priors to estimate the covariate adjusted log-odds of being in each diagnostic category at 5 years as predicted by baseline TAS Test results. The resulting prediction model will estimate posterior highest density intervals for 5-year risk of conversion to MCI and AD using TAS Test results. There is a risk that not all participants classified as MCI will have prodromal AD (as MCI is a heterogeneous group with a variety of causes) but to account for this, we will perform post-hoc analyses of sub-groups with indicators of higher risk, for example, amnestic sub-type of MCI, p-tau181 positive, ApoE4, longitudinal cognitive decline. Data analysis and statistics will be performed in consultation with a biostatistician.

*Descriptive statistics*: The quantitative variables will be described using mean, standard deviation (SD) and range, or median and inter-quartile range if not normally distributed; Qualitative variables will be described using frequency and percentages.

*Test-retest reliability* will be assessed between the first and second completions of TAS Test at home (12 months), and between completions in the research centre and the home (3–6 months). Stability of the TAS Test assessments will be analysed by measuring the intra-class correlation coefficient and by use of Cohen’s kappa coefficient of agreement.

### Data management

All data will be collected online from the participants and stored in databases hosted on the University of Tasmania virtual server farm managed by central IT and backed up daily. Server access is restricted to authorised administrators using Secure Shell and Public Key Infrastructure certificates. Direct access to the databases is limited to system administrators and overseen by designated custodians of the data and will enable access to data in a de-identified fashion to research personnel. Data will be maintained in secure University of Tasmania databases for at least 10 years, and/or until 5 years after the final publication relating to this data, and consent will be sought for this long-term storage as well as linkage to extension projects. We will also be requesting that consent is provided to enable sharing of non-identifiable data with research collaborators external to the University of Tasmania. The study sponsor organisation is the University of Tasmania, Hobart, Australia, 7001. The study management group comprising clinicians, neuroscientists, computer scientists, and a statistician, will meet every month to monitor and discuss the progress of the study, and to address any issues that may arise. Protocol deviations will be reported to the Human Research Ethics Committee in line with local recommendations.

## Discussion

The planned outcome of this project is TAS Test, a new inexpensive computer screening test to estimate the risk of preclinical AD, cognitive decline and AD dementia. If validated, this new scalable tool can potentially transform dementia prevention and research globally. The significant advantages of using a hand-movement based protocol, along with a broad measure of oromotor, visuospatial, memory and language functions, are sensitivity to early preclinical AD and a protocol that has minimal language or cultural barriers. The advantages of using an online test with standard computer equipment is the global reach of the internet crossing geographical barriers and providing accessibility for people in rural and remote communities and those in low-income countries.

Potential risks of the study are acknowledged and strategies to mitigate these are now discussed. There is a risk of inadequate recruitment but we will mitigate this by recruiting existing participants in established research cohorts, namely the longitudinal cohorts of THBP, HBA and ISLAND. There is a risk that TAS Test is not sufficiently accurate but we have mitigated this risk by selecting component test items based on evidence of sensitivity to preclinical AD, combining multiple tests to amplify the multivariable model input data [> 10,000 data points], and electing well-established, transparent statistical modelling approaches that reveal the most discriminatory components of motor-cognitive data, allowing further refinement. We have also devised a study protocol that plans to collect a sufficiently large dataset to employ multiple modelling methods, including feature-agnostic deep learning. A further risk is that participants lack a computer camera to provide video-recorded hand movement data, or do not wish to do so; we have mitigated this risk by recruiting participants from studies that have online assessments already, offering the opportunity for participants to attend the clinical research centre in person if preferred, and providing alternate methods (such as keyboard tapping test and mouse click reaction time tests) within TAS Test that collect movement data. It is conceivable that some participants will not want to know their dementia risk and this will hinder selection of participants for the clinical subset assessments, but we have minimised this risk by recruiting from studies that focus on reducing dementia risk, where participants have already undergone multiple tests of dementia risk. It is also important to acknowledge that, as the bulk of this project relies on self-report of known neurological diagnoses, we have limited ability to make distinctions between other neurodegenerative disorders, including other forms of dementia and disorders which are correlated with dementia (for example, Parkinson’s disease). Finally, there are risks around COVID-19 pandemic restrictions limiting recruitment or progress of the study; as most of the study is based around online movement and cognitive tests that can be completed at home, there are likely to be minimal effects and we will be able to collect blood for p-tau181 levels using personal protective equipment or at a later date.

In summary, this study directly addresses the critical need for population-level screening tests to detect the earliest stages of dementia. ‘*We can’t manage what we can’t measure’* sums up a global critical struggle against the rising tide of dementia and the odds are stacked against drug trials and preventative strategies as AD is typically detected so late in the disease course - when cognitive symptoms arise. Without a screening test to identify preclinical AD risk *early*, our preventive strategies will remain blunted and belated. There is high potential that TAS Test may provide a scalable screening approach, where the test equipment is already in our homes and health centres.

## Supplementary Information

**Additional file 1.** Questionnaire Series of questions that participants are invited to complete at the end of TAS Test.

## Data Availability

The datasets used and/or analysed during the current study are available from the corresponding author on reasonable request.

## Abbreviations

AD: Alzheimer’s disease
AI: Artificial intelligence
Aβ: Amyloid beta
BRAIN: The BRadykinesia Akinesia INcoordination Tap Test
CANTAB: Cambridge Neuropsychological Test Battery
COVID-19: Coronavirus disease 2019
CSF: Cerebrospinal fluid
FT: Finger tapping
HBA: Healthy Brain Ageing Program
ICT: Information and Communication Technology
IPV4: Internet Protocol Version 4
ISLAND: Island Study Linking Ageing and Neurodegenerative Disease
MAPE: mean absolute prediction error
MCI: Mild Cognitive Impairment
MRI: magnetic resonance imaging
ms: milliseconds
NHMRC: National Health and Medical Research Council
PAL: Paired Associate Learning
PD: Parkinson’s disease
PET: Positron emission tomography
pg/ml: picograms per millilitre
P-tau: phosphorylated tau
ROC: Receiver Operating Characteristic
SD: standard deviation
SIMOA: Single Molecule Array immunoanalyser
TAS Test: Tasmania Test
THBP: Tasmanian Healthy Brain Project
USD: United States Dollars
UTAS: University of Tasmania

## Footnotes

1 Tasmania is an island state in South East Australia
