# Supplementary material for: The TAS Test project: a prospective longitudinal validation of new online motor-cognitive tests to detect preclinical Alzheimer’s disease and estimate 5-year risks of cognitive decline and dementia
Source: BMC Neurol. 2022 Jul 18;22:266. doi: 10.1186/s12883-022-02772-5 (PMC9289357; doi:10.1186/s12883-022-02772-5)
Supplement: Supplementary file 1 — Additional file 1. Questionnaire Series of questions that participants are invited to complete at the end of TAS Test. [file 12883_2022_2772_MOESM1_ESM.docx]

1. Were you distracted at any time during testing?

If yes, what test was it during and how did it impact your performance?

Yes

No

Write your comment

2. Were you able to follow the instructions easily?

If no, what would have helped more?

Yes

No

Write your comment

3. How helpful were the text instructions to guide you to complete the test?

0 = not helpful at all; 5 = extremely helpful.

0; 1; 2; 3; 4; 5;

Write your comment

4. How helpful were the video instructions to guide you to complete the test?

0 = not helpful at all; 5= extremely helpful. If 0,1,2, what would have helped more?

0; 1; 2; 3; 4; 5;

Write your comment

5. Are there any specific instructions that you think others would benefit from, that we haven't included?

Write your comment

6. How satisfied are you with the ease of use of this software?

0 = not satisfied at all; 5 = extremely satisfied.

0; 1; 2; 3; 4; 5;

7. Did you enjoy the testing procedure?

Yes

No

Write your comment

8. Did anything bother you about the testing platform? (Text entry)

Write your comment

9. Was the test too long/too short/ about right?

Too Long

Too Short

About Right

10. How did you feel about the recording of videos using your personal camera? (Text entry)

Write your comment

11. What level of computer literacy do you have?

0 = not at all computer literate (no confidence)

5 = extremely computer literate (very confident).

0; 1; 2; 3; 4; 5;

12. Could you see the following element clearly? (select those you could see clearly)

Title

Navigation bar

Test area

Instruction area

Font

All of them

13. Did you feel you had the appropriate hardware (keyboard, mouse, computer to participate in the test? Write down what computer equipment you used for TasTest (laptop, desktop, mouse etc)​

Yes

No

Write your comment

14. What worked well about the TasTest platform?

Write your comment

15. What didn’t work well about the TasTest platform?

Write your comment

16. How could the TasTest platform be improved?

Write your comment

17. Would you prefer to do this computer test at home on your own computer, or in one of our research centres with people on hand to assist you?

At home

At research center

18. Imagine that a BLOOD test is available today, that will accurately tell you what is going on in your brain, and imagine that at the moment, you have no symptoms of dementia or brain disease.

How likely are you to have the blood test for each of the following reasons, on a scale of 1 (very unlikely) to 5 (very likely)?

a.To see how healthy your brain is

0; 1; 2; 3; 4; 5

b.To find out whether you are going to develop dementia in the future

0; 1; 2; 3; 4; 5

c.To help determine whether an intervention or treatment is suitable for you assuming there are effective interventions or treatments available that could stop or slow down progression of the diseases

0; 1; 2; 3; 4; 5

d.To participate in a clinical trial for a new treatment

0; 1; 2; 3; 4; 5

e.Other (free text)

0; 1; 2; 3; 4; 5

Write your comment

19. Imagine that a COMPUTER test is available today, that will accurately tell you what is going on in your brain, and imagine that at the moment, you have no symptoms of dementia or brain disease. How likely are you to have the computer test for each of the following reasons, on a scale of 1 (very unlikely) to 5 (very likely)? (scale 1-5 entry)

a. To see how healthy your brain is

0; 1; 2; 3; 4; 5

b.To find out whether you are going to develop dementia in the future

0; 1; 2; 3; 4; 5

c. To help determine whether an intervention or treatment is suitable for you assuming there are effective interventions or treatments available that could stop or slow down progression of the diseases

0; 1; 2; 3; 4; 5

d.To participate in a clinical trial for a new treatment

0; 1; 2; 3; 4; 5

e.Other (free text)

0; 1; 2; 3; 4; 5

Write your comment

19. Your performance on some sections of Tas Test may be affected by difficulties moving your hands, pain in your hands, or feeling anxious so we would like to know how you were feeling when you started TasTest. This can be taken into account when we analyse your data.

a. Did you have any pain in your hands at the start? Please indicate your level of pain by clicking on the scale of 1 to 10 below, where 10 is severe pain and 1 is no pain at all.

0; 1; 2; 3; 4; 5; 6; 7; 8; 9; 10

b. Did you feel any level of anxiety at the start? Please indicate your level of anxiety by clicking on the scale of 1 to 10 below, where 10 is severe anxiety and 1 is no anxiety at all.

0; 1; 2; 3; 4; 5; 6; 7; 8; 9; 10

20. Have you noticed any change in your WALKING over the past year?

Yes

No

Write your comment

21. Have you noticed any change in your COGNITION (thinking, memory, concentration) over the past year?

Yes

No

Write your comment

22. Have you noticed any change in THE WAY YOUR HANDS WORK over the past year?

Yes

No

Write your comment

23. Are your hands affected by:

Arthritis

Stroke

Parkinson’s

Pain

Other

Write your comment
